# Supplementary material for: Negative Survival Impact of Occult Lymph Node Involvement in Small HER2-Positive Early Breast Cancer Treated by Up-Front Surgery
Source: Cancers (Basel). 2023 Sep 14;15(18):4567. doi: 10.3390/cancers15184567 (PMC10526175; doi:10.3390/cancers15184567)
Supplement: Supplementary file 1 [file cancers-15-04567-s001.zip › cancers-2616302-supplementary.pdf]

Table S1. Regression analyses for adjuvant chemotherapy in all patients.

**Adjuvant Chemotherapy**		**OR**	**CI 95%**	**p**
Grade SBR	1	1		
	2			<0.0001
	3			<0.0001
Age groups	<= 40	1		
	40.1-50	0.498	0.28-0.89	0.019
	50.1-74.9	0.327	0.19-0.56	<0.0001
	>= 75	0.044	0.02-0.09	<0.0001
pT size groups	< 20 mm	1		
	>= 20 mm	2.476	1.74-3.52	<0.0001
LVI	No	1		
	Yes	2.066	1.39-3.06	<0.0001
	Unknown	0.411	0.28-0.61	<0.0001
Subtype	ER-negative	1		
	ER-positive	0.739	0.55-1.00	0.05
pN status	pN0	1		
	pN0(i+)	1.582	0.76-3.28	0.219
	pN1mi	6.727	3.03-14.94	<0.0001
	pN1macro	4.657	3.04-7.13	<0.0001

Table S2. Characteristics of patients with pT1a-b tumors according to adjuvant chemotherapy: univariate analyses.

**pT1a-b**		**No Chemotherapy**		**Chemotherapy**		**Chi 2**
		Nb	%	Nb	%	p
		229	48.3	245	51.7	
Grade	1	50	21.8	12	4.9	<0.0001
	2	129	56.3	120	49.0	
	3	34	14.8	110	44.9	
	unknown	16	7.0	3	1.2	
Age	<= 40	11	4.8	33	13.5	0.007
	40.1-50	50	21.8	54	22.0	
	50.1-74.9	153	66.8	149	60.8	
	>= 75	15	6.6	9	3.7	
Subtype	ER-	72	31.4	119	48.6	<0.0001
	ER+	157	68.6	126	51.4	
Surgery	BCS	174	76.0	176	71.8	0.098
	Mastectomy	46	20.1	65	26.5	
	unknown	9	3.9	4	1.6	
LVI	No	163	71.2	176	71.8	<0.0001
	Yes	7	3.1	43	17.6	
	unknown	59	25.8	26	10.6	
pN	pN0	218	95.2	208	84.9	<0.0001
	pN0(i+) & pN1mi	11	4.8	37	15.1	

Table S3. Positive SN rates in the studies mentionned in the discussion

	ER-positive / HER2-negative	ER-positive / HER2-positive	ER-negative / HER2-positive	triple-negative
Reyal *et al.* [33] (no distinction between pN0(i+), pN1mi and pN1-macro-metastases)	34.7% (439/1,264)	31.6% (25/79)	41.5% (22/53)	20.4% (30/147)
SERC ITC	5.5% (75/1354)	6.67% (9/135)	7.3% (3/41)	8.2% (8/97)
SERC micro-metastases	27.5% (373/1354)	27.4% (37/135)	24.4% (10/41)	29.9% (29/97)
SERC macro-metastases	66.9% (906/1354)	65.9% (89/135)	68.3% (28/41)	61.8% (60/97)
Present study ITC		3% (39/1160)	3% (21/610)	
Present study micro-metastases		7% (85/1160)	5% (33/610)	
Present study macro-metastases		28% (321/1160)	37% (225/610)	

Table S4. lymphovascular invasion rates according to endocrine receptors (ER) status in the studies mentionned in the discussion

	ER-positive / HER2-positive	ER-negative / HER2-positive
ESMO Open 2021 study [39]	22% (3,279/14,655)	35% (926/2,667)
Present study	35.2% (189/537	32.3% (341/1,056)
